# The Silent Revolution of Brewer’s Spent Grain: Meat/Food Innovations Through Circularity, Resource Recovery, and Nutritional Synergy—A Review

**DOI:** 10.3390/foods14193389

**Published:** 2025-09-30

**Authors:** Daniela Tapia, John Quiñones, Ailin Martinez, Erika Millahual, Paulo Cezar Bastianello Campagnol, Néstor Sepúlveda, Rommy Diaz

**Affiliations:** 1 Programa de Doctorado en Ciencias Agroalimentarias y Medioambiente, Universidad de La Frontera, Temuco 4811230, Chile; daniela.tapia@ufrontera.cl; 2 Centro de Tecnología e Innovación de la Carne CTI-Carne, Universidad de La Frontera, Temuco 4811230, Chile; john.quinones@ufrontera.cl (J.Q.); a.martinez26@ufromail.cl (A.M.); nestor.sepulveda@ufrontera.cl (N.S.); 3 Facultad de Ciencias Agropecuarias y Medioambiente, Universidad de La Frontera, Temuco 4811230, Chile; 4 Programa de Doctorado en Ciencias Mención Biología Celular y Molecular Aplicada, Universidad de La Frontera, Temuco 4811230, Chile; 5 Carrera de Biotecnología, Universidad de La Frontera, Temuco 4811230, Chile; e.millahual02@ufromail.cl; 6 Department of Technology and Food Science, Federal University of Santa Maria, Santa Maria 97105900, RS, Brazil; paulo.campagnol@ufsm.br

**Keywords:** by-product, bioactive compounds, meat products, sustainable food

## Abstract

Brewer’s spent grain enhances nutritional quality by increasing fiber and plant-based proteins and reducing the need for synthetic additives. Technologies such as extrusion and fermentation transform BSG into functional ingredients that improve texture and stability. A significant increase in antioxidant capacity was observed in enriched foods; for example, in burgers, BSG improved fiber and protein levels, while decreasing fat and calories without negatively affecting sensory acceptance. In sausages, substituting 5% of pork with BSG achieved acceptance similar to traditional formulations, and hybrid formulations with BSG maintained improved protein content while preserving texture. However, concentrations above 20% may negatively impact sensory and technological properties, by introducing undesirable flavors or altering texture. Thus, BSG is a promising source of high-value functional ingredients that contribute to the circular economy and healthier, sustainable foods. Nonetheless, more in vivo studies are needed to validate the health benefits, understand the interactions in complex matrices, assess the shelf life, and evaluate the long-term sensory perception. The “Silent Revolution” of BSG requires a multidisciplinary approach that integrates science, technology, sustainability, and effective communication with consumers.

## 1. Introduction

Brewer’s spent grain (BSG) accounts for approximately 85% of the total solid waste generated by the brewing industry. It is estimated that for every 100 L of beer produced, approximately 20 kg of fresh BSG or 14 kg on a dry weight basis is generated [1]. Owing to its high content of cellulose, hemicellulose, lignin, and proteins, this by-product has been identified as a valuable resource for food industry applications [2,3]. However, its high moisture content (~70%) and rapid microbial degradation pose significant challenges for its handling and storage [1,4].

Despite these limitations, BSG emerges as a sustainable ingredient with significant economic potential. Recent studies have highlighted its successful incorporation into snacks and bakery products in response to the increasing demand for functional and environmentally friendly foods [5,6,7,8,9]. Concurrently, global demand for meat has increased substantially, driven by population growth and rising average incomes. Projections indicate an additional increase of 48 million tons by 2027, bringing the total to 367 million tons, primarily in developing countries [10]. This trend has fostered a transformation within the meat industry toward more convenient, ready-to-eat formats, adapting to emerging consumption patterns outside the home and fragmented and evolving consumer behaviors [11,12].

In the context of food production, the concept of a silent revolution refers to a gradual and often imperceptible process of social and productive transformation that despite lacking abrupt or confrontational shifts, ultimately reconfigures the agri-food system [13]. Alternative food initiatives—such as sustainable farming practices, short supply chains, and the valorization of by-products—can be understood as “seeds of social change.” These initiatives may not trigger immediate radical upheavals; however, through steady and incremental progress, they quietly reshape the established agri-food landscape. This ongoing evolution fosters a more sustainable and equitable food system, propelled by growing community engagement and a collective commitment.

Within this framework, the incorporation of functional ingredients into processed meat products has emerged as a key strategy to enhance their nutritional, technological, and safety properties [14,15]. These ingredients enable diversification and innovation of product offerings, addressing emerging consumer demands, particularly in terms of flavor, texture, and health benefits. Despite the appreciation for the sensory quality of meat, concerns remain regarding its fat and sodium content and the use of synthetic additives [16,17].

The valorization of agro-industrial by-products rich in bioactive compounds, such as BSG, is emerging as an effective approach to address global challenges related to food security, waste, and the circular economy. Owing to its versatile composition, BSG has garnered interest not only in the food industry but also in sectors such as pharmaceuticals, bioenergy, and nutraceuticals. This review aims to analyze valorization strategies for BSG, focusing on its application in meat products. The first section provides an updated overview of its composition and processing technologies, while the second section concentrates on its incorporation into meat products, hybrid formulations, and analogs, highlighting recent advances and the potential for industrial scale-up.

## 2. Origin and Generation of Brewer’s Spent Grain

### 2.1. Brewing Process, Yield, and Production

Beer has been an integral component of the human diet for millennia and currently represents the most widely consumed alcoholic beverage. Its production is predominantly driven by markets in Asia and the Americas, with China emerging as the leading consumer, accounting for approximately 42,035 kiloliters. This is followed by the United States (20,378 kiloliters), Brazil (14,932 kiloliters), Germany (7827 kiloliters), and the United Kingdom (4587 kiloliters). The global market is estimated to be valued at approximately USD 674 billion [18]. It is projected that this market will exceed USD 797 billion by the year 2032 [19].

Beer brewing involves the transformation of starch-rich grains, primarily barley, through a series of processes including malting, milling, mashing, and boiling. During mashing, malted grains are combined with water in a ratio of approximately 3:1 at temperatures between 60 °C and 65 °C. This temperature range activates enzymes that catalyze the hydrolysis of starches into fermentable sugars [20]. Subsequently, the wort is rinsed to recover residual sugars, resulting in a solid residue composed of husks, pericarp, and endosperm fragments. This by-product is known as brewer’s spent grain or BSG [21].

The clarified wort is then boiled with hops, which are responsible for the characteristic aroma and bitterness. Yeast is then inoculated to facilitate the fermentation, during which sugars are converted into ethanol, carbon dioxide, and secondary metabolites. After fermentation, the product undergoes clarification, maturation, and packaging.

The utilization of BSG can reduce waste management costs and create new revenue opportunities through alternative industrial applications [22]. It is noteworthy that disposal of BSG in landfills can produce up to 513 kg of CO_2_ equivalent per ton, while the treatment of its wastewater generates approximately 83 kg of CO_2_ equivalent per ton [23], highlighting the need for sustainable strategies for its reuse.

### 2.2. Chemical Composition

BSG is primarily composed of fragments of the husk, pericarp, and seed coat of barley, along with remnants of the endosperm and aleurone cell walls [24]. Its chemical composition varies depending on the type of barley, the brewing style, and the processing conditions. Among its most abundant components is total dietary fiber, which ranges from 30 to 70 g per 100 g. This includes insoluble fiber (2.9 to 8.5 g per 100 g) and soluble fiber (29 to 40 g per 100 g) [25]. Hemicellulose, primarily composed of arabinoxylans, ranges from 20 to 40 g per 100 g and is the predominant non-amylose polysaccharide in BSG. This is followed by cellulose (15–30 g per 100 g) and lignin (11–20 g per 100 g) [26,27,28,29].

The protein content ranged from 12 to 31 g per 100 g. The significant fractions identified include hordeins, glutelins, globulins, and albumins, with high concentrations of amino acids such as glutamine, proline, and leucine. However, their limited solubility may restrict certain technological applications [30,31]. The lipid or total fat content ranges from 3 to 13 g per 100 g [32,33]. Notably, triglycerides comprise approximately 55–67% of the lipid fraction, followed by free fatty acids (18–30%). The lipid profile is characterized by a high proportion of linoleic acid (18:2), palmitic acid (16:0), and oleic acid (18:1) [34,35,36].

**Figure 1:**
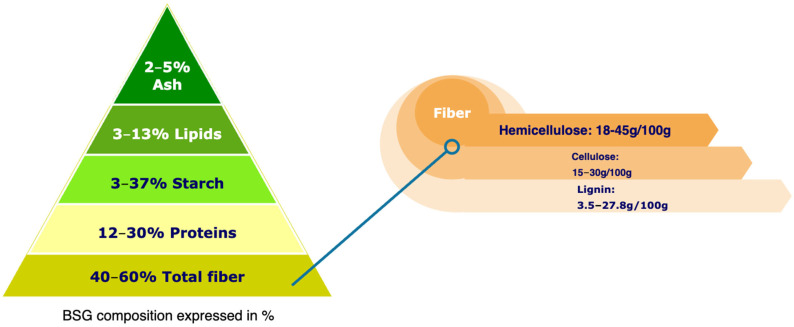
Proximal chemical composition of BSG.

Regarding mineral content, brewer’s spent grain contains approximately 6000 mg/kg of phosphorus, 3600 mg/kg of calcium, and 1900 mg/kg of magnesium [3]. In addition, BSG contains substantial amounts of phenolic compounds, such as ferulic acid (ranging from 1860 to 1948 mg/g) and *p*-coumaric acid (ranging from 565 to 794 mg/g), which contribute to its notable antioxidant capacity [37,38]. The starch content can vary between 3 and 37 g per 100 g, depending on the type of beer and malting conditions [39,40,41]. Additionally, β-glucan levels have been reported to range from 0.74% to 3.5% on a dry basis [42,43,44]. This is summarized in Figure 1.

The observed variations in the composition of BSG can be attributed to factors such as barley variety, the type of beer produced, and even the malting process. Several authors concur with this assertion; for example, Castro & Colpini [45] indicate that minimally modified malts tend to retain a higher amount of residual starch, reaching up to 26.64%. This can be advantageous for certain applications but may also limit the availability of fermentable sugars in the wort. Conversely, extended malting can reduce arabinoxylan levels by degrading cell wall components, enhancing nutrient accessibility. However, this may also lead to the loss of beneficial compounds in BSG, such as dietary fiber [46].

### 2.3. Thermal Processing and Drying

The composition of BSG positions it as a versatile and functional ingredient. However, its effective utilization necessitates overcoming technological challenges related to preservation, extraction of valuable compounds, and functional application. The stabilization of BSG is crucial for its storage and efficient use in food applications. Among the most common preservation technologies are various drying methods, which are essential for reducing microbial growth and enzymatic degradation [4].

Traditional drying methods include freeze drying, freezing, oven drying, superheated steam drying, and rotary drum drying [35,47,48,49,50]. Additionally, combinations of pressing and drying techniques, as well as chemical treatments with organic acids or potassium sorbate, have been employed to enhance preservation. Another technology is freeze drying, or lyophilization, which involves first freezing the BSG and then removing the water through sublimation. This process optimally preserves the nutritional and sensory quality of the product, allowing for the retention of phenolic compounds and antioxidant activity [1,50,51,52]. However, freeze drying is an expensive process that requires significant energy input, limiting its feasibility for large-scale industrial applications [42,53,54].

Among the emerging technologies, infrared drying (IRD) stands out, patented by the ReGrained, Inc. group [55]. This method offers faster drying, greater energy efficiency, and produces products with a crispy texture, toasted aroma, and safe water activity levels [55,56]. Conversely, atomization converts BSG into a liquid suspension that is sprayed into a hot air chamber, resulting in a powdered product that can be produced rapidly [57,58]. This method offers advantages such as short drying times and good solubility of the resulting product. However, high temperatures can negatively impact heat-sensitive compounds, such as polyphenols and certain proteins, which tend to decrease in activity at temperatures above 165 °C [59].

When comparing oven drying with other methods such as freeze drying and atomization, it is evident that oven drying tends to be less effective in preserving the nutritional qualities of BSG. For instance, freeze drying better preserves sensitive compounds such as polyphenols and proteins, whereas oven drying can result in a significant reduction in antioxidant activity and phenolic content. Therefore, oven drying may be a preferable option when seeking a rapid, cost-effective, and easily accessible solution.

### 2.4. Technologies for the Extraction of Bioactive Compounds

BSG is a valuable source of bioactive compounds with potential applications in the food, nutraceutical, and pharmaceutical industries [60,61]. Its complex lignocellulosic matrix contains fibers, proteins, lipids, non-starch polysaccharides, phenolic compounds, and micronutrients [62,63]. The efficient valorization of these constituents necessitates the application of suitable extraction technologies that can effectively liberate, preserve, and concentrate functional ingredients with antioxidant, prebiotic, anti-inflammatory, and other biological activities [64,65,66].

Various methodologies have been developed depending on the target compound, desired yield, chemical stability, and technological requirements of the final product [29,67,68,69,70]. The advantages and limitations of the technologies used in the extraction of BSG are shown in the Table 1. The following sections describe the main extraction strategies for the phenolic, protein, polysaccharide, lipid, and vitamin fractions of brewer’s spent grain.

#### 2.4.1. Phenolic Compounds

Phenolic compounds are secondary metabolites characterized by one or more aromatic rings bearing hydroxyl (-OH) groups. They are widely recognized for their antioxidant capacity and their beneficial effects on human health, including anti-inflammatory, anti-carcinogenic, and cardioprotective properties [71,72]. Their demonstrated anti-inflammatory, antidiabetic, and anticancer activities justify their application in functional foods and nutraceuticals, highlighting their potential to contribute to disease prevention and health promotion [73,74].

BSG is a rich source of phenolic acids, particularly in bound form associated with cell wall structures. Ferulic, *p*-coumaric, and caffeic acids are notable among these. The extraction of these compounds is challenging because of the complex structure of the plant matrix, which limits their release and recovery [50,75]. To release these compounds, various techniques have been implemented, including conventional solid–liquid extraction, microwave-assisted or ultrasound-assisted extraction, and alkaline or enzymatic hydrolysis [75]. However, some methods present limitations: high temperatures can degrade heat-sensitive compounds, and prolonged contact times may reduce extraction efficiency [76]. In response, innovative techniques such as supercritical fluid extraction (SFE), pressurized hot water extraction, and ohmic heating have gained attention because of their efficiency and reduced impact on the structural integrity of the compounds [77,78,79].

The total phenolic content (TPC) in spent brewer’s grain (SBG) varies significantly depending on the extraction method and solvent used, reflecting the advantages and limitations of each approach. For example, Bonifácio-Lopes et al. [80] reported a TPC of 10.44 mg of gallic acid equivalents per gram of BSG when using 80% ethanol and 11.57 mg/g with 60% ethanol, demonstrating the effectiveness of common alcohols as solvents. In addition, solid–liquid extraction (SLE) with the same ethanol concentrations yielded higher values (11.25 mg/g and 13.26 mg/g), showing an improvement in mass transfer and extraction of specific phenols compared to liquid-liquid extraction [80].

In contrast, alkaline hydrolysis has notable limitations, with yields ranging from 1.26 to 4.53 mg/g [81], indicating relatively low efficiency compared to other methods and restricting its large-scale application without further optimization of the process. In contrast, microwave-assisted extraction (MAE) has shown significantly higher yields, reaching values between 15 and 20 mg/g [82], positioning it as an advantageous technique compared to conventional methods. However, its scalability is limited by equipment costs and challenges in achieving uniform heat distribution, which can affect reproducibility in industrial processes.

Among the most efficient technologies, subcritical water extraction (SCW) at 185 °C stands out for its high efficiency, with a CFT close to 33 mg/g [83], highlighting its ability to break down the BSG matrix and effectively release phenolic compounds. However, this technique requires equipment capable of operating at high temperatures and pressures, thereby increasing the operational complexity and associated costs. In contrast, aqueous extractions with a variable pH allow the influence of pH on phenol release to be studied; however, their advantages are not clearly defined, as the reported CFT values are very low (0.18–2.76 mg/g) [84], suggesting limited effectiveness and critical dependence on pH to obtain useful results.

Deep eutectic solvents (DESs) have emerged as promising alternatives, in some cases outperforming traditional methods such as methanol–water extraction and are presented as sustainable and environmentally friendly options [85]. However, their main limitation is the need for industrial-scale validation to confirm their viability and scalability in commercial processes. Emerging and hybrid technologies offer advantages such as higher yields and better preservation of the functional activity of phenols, with the potential to combine the benefits of different approaches to improve efficiency, selectivity, and sustainability. However, the lack of validation or implementation at an industrial scale remains a major challenge, requiring further studies to ensure their commercial viability, safety, and sustainability on a large scale [84,85].

Various studies have explored innovative strategies to optimize the extraction of bioactive compounds to enhance the yield, selectivity, and sustainability of the process. Connolly et al. [84] employed aqueous extracts with varying pH levels, resulting in notably low TPC values ranging from 0.18 to 2.76 mg/g of BSG. Additionally, microwave-assisted extraction using deep eutectic solvents has shown promising results, surpassing traditional methods such as methanol–water extraction [86]. These green solvents represent a sustainable alternative; however, they still require validation at an industrial scale. In this context, the efficiency of phenolic compound recovery of BSG is critically dependent on the extraction method, operational conditions (solvent, temperature, time), and the nature of the target compound. Emerging and hybrid technologies offer significant advantages over conventional methods, enhancing both extraction yield and the preservation of functional activity.

#### 2.4.2. Bioactive Proteins and Peptides

Proteins are essential macromolecules that are fundamental to cellular structure and function. They catalyze enzymatic reactions, regulate metabolic processes, and participate in immune defense mechanisms [86,87]. BSG contains a substantial proportion of proteins, with concentrations ranging from 12% to 30% on a dry basis, surpassing many other lignocellulosic agro-industrial residues [1]. This variability depends on the barley type, additives used, and brewing process employed during beer production [88].

The most abundant protein fractions in BSG are hordeins, glutenins, globulins, and albumins [89]. A predominance of amino acids, such as glutamine and proline, along with leucine, phenylalanine, valine, isoleucine, and threonine, have been observed in the protein extracts [30,62,90,91]. Special attention was given to lysine, an essential amino acid often limited in cereals, which is present in concentrations of approximately 3%. It fulfills about 80% of the daily human requirement [90].

The extraction and valorization of proteins from BSG focuses on their transformation into bioactive peptides through enzymatic hydrolysis. In particular, the use of proteases such as alcalase has significantly improved the solubility, stability, and antioxidant capacity of protein extracts [92,93]. The peptides produced exhibit significant functional properties, including antioxidant, immunomodulatory, antihyperglycemic, antihypertensive, and antimicrobial effects [94].

In the industrial sector, a notable example is the development of commercial products such as EverPro^®^ by EverGrain (Wilmington, DE, USA). This product offers protein concentrates with a protein content of >85%. It is obtained through enzymatic hydrolysis followed by micro- and nanofiltration, producing highly soluble ingredients intended for use in functional beverages and foods [94,95]. Peptides derived from BSG have potential as functional ingredients in both human foods and animal supplements. However, additional studies, particularly in vivo experiments and clinical trials, are necessary to confirm their biological efficacy, safety, and bioaccessibility under actual physiological conditions [36,96].

#### 2.4.3. Polysaccharides and Dietary Fiber

Non-starch polysaccharides (NSPs) are complex carbohydrates that are indigestible by human enzymes and are composed of long chains of monosaccharides linked by glycosidic bonds that are different from those found in starch. Their primary function is structural, forming part of the cell walls in plants, and they constitute a key component of dietary fiber [97,98,99]. BSG contains significant amounts of NSPs, notably β-glucan (1,3-1,4-β-D-glucan) and arabinoxylans. Both exhibit valuable techno-functional and nutritional properties, particularly because of their prebiotic effects and ability to modulate the gut microbiota.

Enzymatic hydrolysis is one of the most widely used techniques for the degradation of polysaccharides, such as cellulose and hemicellulose, present in BSG. Mussatto et al. [1] demonstrated that the use of specific enzymes, such as cellulases and xylanases, allows for the efficient release of fermentable sugars and soluble dietary fiber. This technique is highly selective and does not generate toxic by-products, making it ideal for applications in the food and biofuel industries.

Physical and chemical pretreatments are essential for modifying the lignocellulosic structure of CSB and facilitating polysaccharide extraction. For example, microwave-assisted pretreatment (MAE) has shown promising results in this regard. Alkaline pretreatment using sodium hydroxide (NaOH) or alkaline hydrogen peroxide (AHP) is effective in reducing lignin content and improving the accessibility of polysaccharides, as highlighted by [100]. Although this method is economical, it generates corrosive by-products.

Subcritical water extraction (SCW) is an emerging technology that operates at high temperatures and pressures to break down the lignocellulosic matrix of the BSG. Kumar et al. [57] reported significant yields of polysaccharides and dietary fiber, highlighting its efficiency in releasing bioactive compounds. However, this technique requires specialized equipment and incurs high operating costs, which limit its large-scale application.

Microbial fermentation is a sustainable alternative for BSG processing. Microorganisms, such as fungi and bacteria, can degrade polysaccharides and produce compounds of interest, such as bioethanol, organic acids, and enzymes. Aliyu & Bala [47] showed that this technique is viable to produce biofuels and bioproducts, although its efficiency depends on the culture conditions and the microbial strain used.

Deep eutectic solvents (DES) have emerged as green and sustainable alternatives for extracting polysaccharides and dietary fiber from BSG. Procentese et al. [101] have shown that these solvents improve the solubility and selectivity of the compounds of interest, in some cases outperforming traditional methods such as methanol–water extraction. However, their implementation on an industrial scale requires validation and parameter optimization.

Finally, emerging and hybrid technologies, notably as the combination of ultrasound with enzymes or the integration of physical and biological processes, have shown significant advantages in terms of efficiency and sustainability of extraction. Moreira et al. [82] have demonstrated that ultrasound improves the efficiency of enzymatic hydrolysis, thereby reducing processing time and increasing sugar yield.

Overall, the processing of polysaccharides and dietary fiber from BSG has advanced significantly owing to the development of various technologies. While methods such as enzymatic hydrolysis and chemical pretreatments are widely used, emerging techniques such as SCW and DESs offer advantages in terms of sustainability and efficiency. However, industrial-scale implementation remains a challenge, particularly in terms of cost and technical validation. Future studies should focus on optimizing these processes to maximize their commercial viability and contribute to a circular economy.

##### β-Glucans

The β-glucans in BSG are composed of β-D-glucopyranose residues linked primarily by β (1→4) bonds, which are interrupted by β (1→3) linkages. This structural arrangement directly influences their solubility and viscosity [102,103]. This polysaccharide has been associated with beneficial metabolic effects, such as the reduction in postprandial glycemic and insulinemic responses and has been validated by regulatory agencies such as the European Food Safety Authority (EFSA) [104,105,106].

The functional activity of β-glucan depends on its molecular weight (MW) and degree of polymerization (DP). It has been reported that MWs exceeding 50 kDa are more effective in exerting hypocholesterolemic and prebiotic effects [94,95]. Recently, Kobelev et al. [104] employed enzymatic extraction using cellulases and xylanases, resulting in β-glucans and arabinoxylans with molecular weights between 200 and 300 kDa and purity levels exceeding 85%, making them suitable for nutraceutical applications. However, the scalability of this method is limited by the high cost of enzymes and the need for precise control over operational parameters.

In contrast, Steiner et al. [43] applied a simple hydrothermal treatment (80–100 °C for 1–3 h) and achieved high yields (~20%) but resulted in lower molecular weights (50–100 kDa), which are more suitable for basic, low-cost food products. Jantason et al. [42] combined hot water extraction with enzymatic treatment, optimizing the conditions using response surface methodology (RSM). This approach achieved an extraction yield of 18%, with molecular weights between 150 and 250 kDa and purity levels exceeding 90%. Thus, this strategy represents an optimal balance between product quality and technical feasibility.

From an analytical perspective, Zielke et al. [44] characterized β-glucans from BSG using asymmetrical flow field-flow fractionation (AF4) and observed their association with proteins, which influences their solubility and rheological behavior. These findings are crucial for optimizing the extraction conditions and formulation strategies for functional ingredients.

##### Arabinoxylans

Arabinoxylans (AX), composed of xylose and arabinose, are dietary fibers found in BSG. Their structure features a β-(1→4)-xylopyranose backbone with arabinofuranose side chains that can be esterified with ferulic acid. The structural variability depends on the plant source and extraction method [107,108,109,110]. The recovery of AX from BSG is challenging because of its association with cellulose microfibrils via hydrogen bonds and diferulic acid cross-links. Enzymatic hydrolysis alone is often insufficient to disrupt these bonds, limiting the extraction efficiency [109].

The potential of BSG as a source of AX has been extensively explored because of its industrial viability and functional benefits. Recent studies have demonstrated that AX from BSG can modulate gut microbiota, promote beneficial bacteria such as Bifidobacterium and Lactobacillus, and increase short-chain fatty acid production [108,111]. These properties highlight AX’s role as a prebiotic ingredient, although further in vivo validation in humans is necessary.

Several innovative extraction techniques have been developed. Methods like particle size reduction, enzymatic treatment, solid-state fermentation, and hydrothermal processes have been shown to enhance AX solubility, yield, and functional properties, including antioxidant capacity [107,110,112,113]. These approaches are scalable but require optimization to reduce costs and processing time. Chemical modifications, such as carboxymethylation, improve functional traits like emulsification capacity, broadening industrial applications [104,108,109,111]. Physical treatments, including ultrasonication and nixtamalization, have achieved recovery rates exceeding 70%, offering practical options for large-scale implementation [108,109,111].

The valorizing of BSG as a source of AX involves a spectrum of strategies tailored to different objectives—ranging from cost-effective hydrothermal methods to high-value enzymatic or chemical modifications. Integrating these processes within a circular economy framework requires ongoing optimization, health impact assessments, and considerations of environmental and economic sustainability. Future research should focus on in vivo validation, industrial scaling, and life cycle analysis to fully realize the potential of AX from BSG in food and nutraceutical applications.

#### 2.4.4. Lipids

Recent research has placed a particular emphasis on the recovery of high-value lipid fractions from BSG. This by-product contains a complex matrix of neutral and polar lipids, whose structural and bioactive properties may offer significant nutritional and technological advantages in advanced formulations [108].

Conventional solid–liquid extraction techniques, based on organic solvents of varying polarity, remain the initial approach for isolating triacylglycerols, phospholipids, and free fatty acids. Additionally, alkaline hydrolysis protocols and enzymatic processes have demonstrated effectiveness in releasing lipid fractions bound to proteinaceous or fibrous matrices [109]. However, the selectivity towards specific compounds and the energy efficiency of these methods necessitate optimization to ensure the preservation of the functional integrity of the extracted lipids.

To overcome these limitations, advanced extraction methods have been incorporated, including supercritical fluid extraction (SFE) using CO_2_, microwave-assisted extraction, and ultrasound-assisted extraction. These technologies facilitate the disruption of cellular structures and enhance lipid solubilization, resulting in reduced solvent consumption and shorter processing times. Specifically, CO_2_-based SFE, optimized at 313 K and 35 MPa on ground BSG, has demonstrated not only maximum yield in recovering neutral fractions but also the preservation of sensitive polyunsaturated fatty acids [75,111].

BSG exhibits an approximate lipid content of 10–12%, comprising a mixture of triacylglycerols, phospholipids, and free fatty acids. Among these, linoleic and oleic acids are particularly prominent, along with small proportions of short-chain fatty acids that may impart emulsifying and nutraceutical properties to the extracts. The prior application of supercritical fluid extraction (SFE) has also been described as an effective pretreatment, enhancing subsequent enzymatic hydrolysis and facilitating the production of low-molecular-weight lipids with specific functional activities [83,114].

The lipid fractions recovered from brewer’s spent grain (BSG) offer a diverse range of applications in high-value industries. In the food sector, they serve as emulsifying ingredients or sources of essential fatty acids; in cosmetics, their emollient and antioxidant properties are exploited; and in pharmaceutical formulations, they can be incorporated as lipid carriers for active compounds. Additionally, their use in packaging materials or biodegradable coatings introduces new opportunities toward circular and sustainable processing approaches [114].

Recent research has explored various techniques for extracting valuable compounds from BSG, a significant by-product of the brewing industry. Conventional methods include solid–liquid extraction using organic solvents, as well as alkaline and enzymatic hydrolysis processes [109]. Advanced techniques such as pressurized fluid extraction, supercritical CO_2_ extraction, microwave-assisted extraction, and ultrasound-assisted extraction have demonstrated significant promise in enhancing extraction yields and process efficiency [75,109]. Supercritical CO_2_ extraction, optimized at 313 K and 35 MPa using ground BSG, has demonstrated economic feasibility [111].

#### 2.4.5. Vitamins and Minerals

Vitamins, essential organic compounds, function as coenzymes in critical metabolic processes, including energy production, tissue synthesis, and antioxidant defense mechanisms [115]. Recent studies have highlighted cereals and their derivatives, such as BSG, as promising sources of B-complex micronutrients. Hassani et al. [116] identified that germinated grains significantly increase the concentration of water-soluble vitamins, an effect attributed to enzymatic activation during germination. For example, in beer, Hucker et al. [117] reported variations in thiamine (B_1_: 0.02–0.15 mg/L) and riboflavin (B_2_: 0.03–0.12 mg/L), depending on the raw materials and malting conditions.

According to Diósi and Nagy [118], for every 100 g of dry BSG, there are between 0.8 and 1.2 mg of B vitamins (on a dry basis), with notable levels of thiamine (B_1_: 0.4 mg/100 g) and riboflavin (B_2_: 0.3 mg/100 g), which are essential for energy metabolism and neuronal function. However, fat-soluble vitamins such as A, D, and E are present in marginal concentrations (<0.3 mg/100 g), limiting their direct nutritional contribution. In contrast, van Bokhorst-van de Veen et al. [119] demonstrated that fermentation with Propionibacterium freudenreichii can increase vitamin B_12_ content in BSG from 0.2 µg/100 g (raw) to 2.8 µg/100 g, along with a 15% improvement in protein digestibility and a 40% reduction in phytates, thus enhancing its nutritional value.

The bioaccessibility of vitamins varies dramatically depending on the food matrix. Farcaș et al. [63] reported in vitro bioavailability values of 72.45% for thiamine, compared to 16.47% for pyridoxine (B_6_), underscoring the importance of optimizing extraction methods. Techniques such as enzymatic hydrolysis (using xylanases and cellulases), ultrasound, and supercritical fluids have demonstrated effectiveness in releasing bioactive compounds, with critical parameters including temperature (37–60 °C), pH (4.0–6.5), and liquid-to-solid ratios (10:1–20:1) [114,120]. Additionally, extracts obtained from BSG using gentle methods contain non-cytotoxic phenolic compounds (e.g., catechin: 12 mg/g; vanillin: 0.8 mg/g) at concentrations ≤1 mg/mL, suitable for functional applications [80].

Therefore, the extraction methodologies or techniques employed to obtain various bioactive compounds significantly influence the retention of micronutrients. Afify et al. [121] observed a reduction of 40–60% in total phenolics, flavonoids, and vitamin E following grain maceration. Conversely, Hassani et al. [116] highlighted that higher degrees of maceration (35–41%) can increase the content of riboflavin (B_2_) and prebiotic arabinoxylans, suggesting a balance between thermal losses and enzymatic activation.

## 3. Use of BSG in Food Production

**Figure 2:**
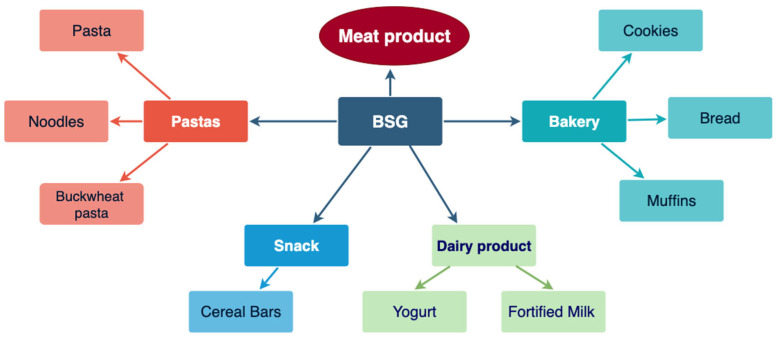
Applications of BSG in food production.

The research has encompassed a wide range of food products, exploring various methods of utilizing BSG with diverse objectives. The incorporation of BSG into bread is a major focus due to its high global consumption. Studies primarily aim to enhance the nutritional value, particularly by increasing fiber and zinc content, as well as improving the sustainability of baked goods [7]. Various methods have been explored for incorporating BSG, such as partial substitution of flour with dried BSG flour [122,123], as shown in Figure 2.

The addition of BSG in the form of a fermenting agent has also been investigated as a method to enhance its functional properties and facilitate its integration into various food products [124], or direct inclusion into the dough. The studied incorporation levels vary considerably, ranging from low levels of 2–8% [123] and up to 10% substitution aimed at enhancing nutritional content and overall product quality [125], and at higher levels, up to 20% in steamed bread or 40% and 60% in chocolate cakes, to achieve a greater nutritional impact [7,122]. Additionally, the impact of BSG derived from different styles of craft beer and non-conventional brewing processes has been evaluated regarding the sensory properties, composition, and nutritional value of bread [126,127,128].

In pasta products, BSG is primarily used to enhance the protein and fiber content, while also incorporating antioxidants and potentially reducing the glycemic index. Various forms of BSG have been investigated, including direct BSG incorporation, to evaluate their effects on product quality and nutritional profile [5,129,130], fermented BSG [129], BSG bioprocessed [131], derived from BSG, EverVita Fiber (EVF), and EverVita Pro (EVP) were developed by Anheuser-Busch InBev (Leuven, Belgium), where EVF is BSG-enhanced to have a higher fiber content, while EVP is enriched to have a higher protein concentration. Ingredients derived from brewer’s spent grain (BSG) are enriched in fiber (EVF) or protein (EVP) [132]. The levels of direct incorporation of BSG in noodles and pasta vary, with percentages of 5%, 10%, 15%, and 20% in noodles [5], and 5% and 10% in general pasta [130] having been studied. Specific concentrations, such as 5 g and 10 g of BSG per 100 g of semolina, have been used in buckwheat pasta (Neylon et al. 2021), as well as a 10% inclusion of barley-derived BSG in pasta formulations [133].

The incorporation of BSG into cookies aimed to improve their physicochemical, nutritional, and sensory profiles. Studies have investigated how the composition and percentage of BSG influence chemical and sensory properties, with substitution levels of flour ranging from 30% to 40% [6]. Additionally, the effects of partial flour substitution on physical, chemical, and sensory properties at levels up to 75% have been investigated [134]. Furthermore, the impact on postprandial glycemic response in individuals with metabolic syndrome has been evaluated using a 30% substitution of wheat flour with BSG treated by autoclaving or fermentation [135]. The use of fermented solid-state BSG has also been studied in biscuits at levels of 15% and 30% to improve the nutritional profile, antioxidant properties, and digestibility [136]. In bread biscuits, a 30% partial replacement of flour with BSG was explored, enriched with oats, aiming to valorize the by-product and develop functional foods [128].

In this context, the fortification of muffins with BSG primarily aims to enhance their nutritional and functional properties, such as antioxidant and antidiabetic activities, as well as increasing fiber and protein content. Different forms of BSG are employed, including hydrolyzed BSG protein at levels of 2%, 4%, and 6% [52], enzymatically hydrolyzed BSG incorporated as a substitution in the mixture at 5%, 10%, and 15% [137], or dried BSG flours replacing part of the flour at 10%, 15%, and 20% [8].

The incorporation levels of BSG vary depending on the formulation, with studied percentages of 8.5%, 12.7%, and 21.2% in honey-based bars, and 3.9%, 7.7%, and 15.5% in chocolate-based bars [138]. Additionally, malt bagasse has been used at levels of 19% and 24% to develop high-fiber bars [139]. Conversely, studies have suggested that increasing the concentration of BSG in flour blends leads to significant improvements in protein content, dietary fiber, lipids, and ash levels [140]. Specifically, incorporating BSG at levels ranging from 5% to 40% in formulations such as bread, muffins, cookies, and pasta results in increased content of proteins (2–25%), ash (2.5–65%), and dietary fiber.

Regarding protein content, although a general increase is observed with BSG addition, specific studies have revealed variable effects depending on the inclusion level and product type [140]. Some research found no significant differences in protein content at up to 10% BSG in bread, breadsticks, and pizza preparation [141], 15% in muffins [137], 30% in bread [9], and 5–20% in pasta formulations [142]. However, significant differences were reported when inclusion exceeded 10% in other studies [8,140,143].

The particle size of BSG flour also plays a critical role in protein content. Czubaszek et al. [140] reported that cookies prepared with medium and coarse particles (425–850 µm) contained lower protein levels (13–21%) compared to those made with fine flour (212–425 µm). In contrast, Öztürk et al. [144] obtained a protein content of 31% for cookies made with fine flour. Similarly, BSG flour exhibited a decrease in protein content from 28.2% at a particle size of 0.21 mm to 22.7% at 0.60 mm [145]. The influence of particle size on protein content remains underexplored.

The addition of BSG has an even more pronounced impact on dietary fiber content [140]. Studies have shown that adding 15–20% BSG in rye or wheat bread and pasta increased fiber content by 4 to 5.5 times [131,140,146]. In the same way, Fărcaş et al. [35] found that incorporating 5% BSG into wheat bread doubled the dietary fiber, while a 20% addition increased it fivefold. Comparable improvements in fiber content have been observed in muffins [8,137], bread [140,146], and pasta formulations [142,147]. Fermentation and enzymatic treatments further enhance fiber content [129,148]. The addition of spray-dried fermented BSG with LAB increased fiber from 2 to 6.5 g/100 g [129].

Similarly, enzymatic hydrolysis of BSG-based bread resulted in fiber levels 2.5 times higher compared to control bread [148]. Microbial enzymes and enzyme blends present in fermented BSG may contribute to the solubilization of dietary fiber, especially arabinoxylans [129]. As with protein, BSG particle size influences fiber levels in enriched products; cookies made with coarser BSG particles (425–850 µm) showed higher total dietary fiber than those with fine (<212 µm) or medium (212–425 µm) sizes [144]. Fine BSG particles contain less fiber than medium or coarse sizes [144]. Additionally, the addition and modification of fiber significantly affect food texture, stability, and water retention during processing, along with health benefits such as reduced digestibility and improved gut health [144,149,150].

In another context, it has been reported that incorporating BSG into various food formulations results in notable enhancements in antioxidant profiles and phenolic compound content. Specifically, pasta, cookies, muffins, and beverages enriched with BSG demonstrated increases in total phenolic content, total flavonoids, and antioxidant capacity measured through assays such as ABTS and DPPH.

For example, in muffins, the addition of 20% BSG flour increased total phenolics by 76% and enhanced antioxidant activity (RSA: 185%) [8]. In cookies, using 30% BSG raised antioxidant activity by 28% (ABTS) and increased DPPH radical scavenging capacity sevenfold [143], while another study noted a fourfold increase in TPC and a 19% rise in antioxidant activity [151]. In pasta, incorporating 20% BSG improved overall antioxidant capacity by approximately 13% [142]. Bioprocessed BSG (FBSG, 15%) stood out with a 70% increase in TPC and improvements of 23–40% in antioxidant activity [131]. Lastly, extruded snacks containing 40% BSG showed dramatic increases: 4–7 times in TPC, 19 times in DPPH radical scavenging, and 5 times in FRAP assay results [152]. These findings underscore the potential of BSG as a functional ingredient to enrich foods with antioxidant and nutraceutical properties.

Specifically, significant increases in particular phenolic acids have been observed. Cookies enriched with up to 30% BSG showed notable elevations in phenolic acids such as *p*-coumaric acid (from undetectable levels to 709.3 µg/g) and ferulic acid (from 419.2 to 3054.4 µg/g) [143]. Similar increases in ferulic acid content were reported in bioprocessed bread with FBSG, rising from 1.32 to 2.60 mg/100 g [153]. The main factor contributing to the high soluble ferulic acid levels in these breads was enzymatic bioprocessing; enzymatic hydrolysis and fermentation of BSG, along with the presence of acids such as *p*-coumaric, ferulic, sinapic, and caffeic acid, as well as bioactive peptides, contributed to increased phenolic activity and antioxidant capacity [8,131,151].

Ferulic acid is particularly significant due to its antioxidant activity comparable to vitamin C, which aids in food preservation by preventing oxidation [151]. As evidenced in these studies, particle size notably influences the final composition of enriched products. Finer particles favor higher protein content [145,154], whereas coarser particles optimize dietary fiber content [154]. This highlights the importance of developing milling techniques to adjust granulometry for maximizing specific nutrients based on the type of food product.

Enzymatic hydrolysis and fermentation with lactic acid bacteria (LAB) have proven effective tools for increasing the solubilization of bioactive compounds such as arabinoxylans and phenolic acids [129,148]. However, scaling these processes to industrial levels presents challenges, particularly regarding costs, control of fermentation parameters, and maintaining functional properties in final products.

Despite the nutritional and functional benefits of BSG, its inclusion in food products can present sensory and technological challenges, especially when used in concentrations above 20%. One of the main problems is the alteration of texture and stability, as BSG can affect the structure of breadcrumbs, reduce their volume, and modify their alveolar architecture [155]. In addition, its water retention capacity can negatively influence the process stability, particularly in baked and extruded products. In terms of organoleptic profile, the incorporation of WBS can introduce bitter or earthy flavors, as well as color changes, which could limit its acceptance by consumers [149,156,157]. To mitigate these undesirable effects, strategies such as encapsulation of active compounds or mixing with other ingredients have been proposed, which could improve the sensory acceptance of BSG-enriched products, including bread.

In addition to these challenges, the consumption of SGF-fortified foods offers significant health benefits. First, the insoluble fiber present in the SGF acts as a prebiotic, promoting colonic fermentation and modulating the gut microbiota, which contributes to improved digestive health. A recent clinical study evaluated the effect of cookies formulated with BSG on gastrointestinal well-being, demonstrating that these cookies are not only safe and well-tolerated but also significantly improve intestinal health compared to commercial cookies [69]. Second, BSG can help regulate postprandial glycemic responses. A clinical study with 40 normoglycemic subjects demonstrated that a dietary supplement based on BSG extract, rich in soluble dietary fiber and bioaccessible ferulic acid, restored baseline blood glucose and insulin levels at 120 min, thus reducing postprandial increases in subjects with mild insulin resistance [158]. In addition, BSG-enriched cookies showed higher nutritional values than commercial wheat-based cookies, suggesting that they may be an effective option for regulating the glycemic response in individuals with metabolic syndrome [144].

However, although the prebiotic and antioxidant effects of BSG are promising, the clinical evidence remains limited. In vivo studies and controlled trials are essential to confirm its impact on metabolic health and gut microbiota. The interactions between BSG and other food ingredients during processing (flours, emulsifiers) have not been thoroughly explored, particularly regarding their effects on texture, stability, and nutrient release. While BSG is an abundant and cost-effective by-product, implementing bioprocessing and milling techniques at an industrial scale requires comprehensive feasibility analyses considering economic viability and environmental sustainability. The development of BSG-enriched products offers an opportunity to promote sustainability within the food industry by valorizing waste by-products of brewing and enhancing nutritional profiles. Nonetheless, the successful integration of this functional ingredient depends on a balanced approach that combines technological innovation, sensory optimization, and scientific validation of health benefits.

## 4. Development of Meat Products Incorporating BSG

The meat industry is increasingly interested in integrating functional ingredients to enhance the nutritional profile of its products. In this context, BSG has emerged as a promising additive, notable for its high fiber and protein content. For example, Kim et al. [159] evaluated the effect of dietary fiber extracts from BSG on the quality characteristics of chicken burgers. After extraction, the total fiber content of the extracts increased from 58.11% to 68.57%, and these extracts were incorporated into the burgers at concentrations of 0, 1, 2, 3, and 4%. The results indicated that a 3% dietary fiber extract from BSG could serve as an effective source of fiber to improve the quality properties of chicken burgers.

In another study, the stability of smoked sausages was evaluated over a storage period of 7 and 15 days, along with their physicochemical and microbiological characteristics, using different concentrations of brewer’s spent grain (1%, 3%, 5%, and 6% *w*/*w*). The results showed that storage time had a significant impact on the physicochemical and microbiological properties of the smoked sausages. Among the samples tested, the smoked sausage containing a mixture of 3% BSG and 8% fungi was the most preferred by the panelists [160].

A preliminary study conducted by Fogarasi et al. [161] evaluated the substitution of different levels of pork meat with brewer’s spent grain (5%, 10%, 15%) in smoked sausages to assess consumer acceptability compared to 100% pork sausages. The results indicated that sausages containing 5% BSG received a similar level of acceptance as the control, which was made solely with pork meat. Saraiva et al. [162] assessed the antioxidant activity, physicochemical properties, sensory attributes, and cooking performance of burgers enriched with BSG. The inclusion of BSG increased the fiber and protein content while reducing fat and caloric value. Although the hardness and chewiness values rose, the antioxidant activity of the burgers improved significantly. Importantly, consumers did not perceive statistically significant differences (*p* < 0.05) between formulations with and without BSG, indicating that the product was well accepted.

A study by Campos et al. [163] evaluated the substitution of pork meat with BSG and concluded that this did not significantly alter texture properties, nor did it prevent oxidation in the sausage or exhibit antimicrobial activity. However, an increase in dietary fiber and protein content was observed, contributing to an improved nutritional profile of the product. The total phenolic compounds were also determined, which may indicate a potential antioxidant capacity, inferred from the measured phenolic content.

As previously reviewed, BSG has been highlighted as a rich source of dietary fiber, proteins, and bioactive compounds across a variety of food products. Particularly in the context of meat products, incorporating BSG into their development contributes to creating healthier and more sustainable foods. For example, in plant-based products, the partial substitution of meat with BSG, broccoli, and insects in the production of hybrid sausages has achieved a significant increase in protein content, while maintaining a texture similar to that of traditional sausages and ensuring sensory acceptance [164]. This demonstrates that BSG can be used in hybrid formulations to balance nutritional quality and functionality. Similarly, in products such as burgers and frankfurter-type sausages, replacing fat with BSG has enabled caloric reduction, increased fiber content, and improvements in textural parameters without compromising sensory quality [162,165] (Table 2). These findings are especially relevant for the development of low-fat meat products aligned with current consumer demands for health and sustainability.

Brewer’s spent grain not only enriches products nutritionally but also enhances key technological properties such as water retention capacity (WRC) and texture. In chicken burgers, the addition of BSG fiber increased moisture retention and resulted in a firmer texture, especially during baking in a convection oven [159]. In low-fat chicken sausages, BSG fiber also contributed to improved cohesion and a better textural profile [167]. Furthermore, incorporating BSG into traditional sausages at concentrations of 2–4% helped maintain physicochemical stability and extended shelf life up to 21 days, highlighting its potential as a stabilizing agent [156].

The inclusion of BSG in meat analogs and extruded snacks has shown significant progress, as it improves the fibrous texture and nutritional profile, making these products more similar to meat in terms of cohesion and bite [168,169]. Likewise, blending BSG with flours derived from poultry meat enabled the development of high-protein, high-fiber snacks with good sensory acceptance, demonstrating its versatility in high-temperature systems [155].

Studies by Curutchet et al. [170] emphasize that communicating the environmental benefits of using BSG in enriched products can significantly increase consumer acceptance. The willingness to purchase fiber-enriched burgers made from BSG extracts varied considerably depending on label information and brand. All consumers showed interest in buying these products if they were from a leading brand; additionally, 85% expressed they would also purchase them if the brand was artisanal. While brand influence significantly affected purchase intention, the results also reveal a segment of consumers with a positive attitude towards the concept of a “sustainable burger,” which the food industry could leverage to position such products [170].

Partial replacement of meat or fat with BSG, as in hybrid sausages and frankfurters [164,165], helps reduce the carbon footprint associated with animal production—a particularly relevant action given that the meat industry is one of the major greenhouse gas emitters. However, BSG can introduce undesirable flavors or textures in some products, especially at high concentrations (>20%). For example, in extruded snacks and meat analogs, balancing BSG functionality with sensory perception is essential to ensure commercial success [155,168].

Although technologies such as extrusion and microencapsulation have proven effective for incorporating BSG into food products, scaling up faces technical and economic challenges that have not yet been fully evaluated. Optimization of processes like milling and enzymatic treatment requires further research to maximize BSG’s functionality without significantly increasing costs. Additionally, while BSG improves texture and nutritional content in certain products, its interactions with animal proteins and emulsifiers have not been sufficiently studied—an aspect critical for ensuring stability and quality in complex products like sausages and burgers. Finally, the potential health benefits of BSG, such as improved digestion and modulation of gut microbiota, remain largely unexplored within the context of meat products. Long-term clinical studies are needed to validate these effects and to investigate possible interactions between its bioactive components and meat matrices.

In recent years, the research on BSG valorization has shifted toward greener, more integrated extraction strategies that maximize yield while minimizing environmental impact. Subcritical water extraction (SWE) has emerged as a leading approach for phenolic recovery, offering tunable extraction parameters to balance yield and bioactivity while avoiding organic solvents [83]. This method also facilitates partial solubilization of polysaccharides, although cellulose purity and process scalability remain areas for further optimization.

Natural deep eutectic solvents (DESs/NADESs), composed of biodegradable components such as choline chloride and organic acids, have shown high selectivity for phenolics and proteins, particularly when coupled with ultrasound-assisted extraction (UAE) or microwave-assisted extraction (MAE) [85,171,172]. These hybrid approaches reduce extraction time, enhance mass transfer, and operate under mild conditions, preserving thermo-labile compounds. Adjusting DES viscosity with controlled water addition has been identified as critical for large-scale processing [173].

Parallel advances have been made in biorefinery cascade processes that valorize wet BSG without energy-intensive drying. Sequential fractionation (often combining enzymatic hydrolysis with hydrothermal treatment) has achieved protein yields of up to 65% and oligosaccharide recovery above 90%, while preserving antioxidant activity [174,175]. This integrated approach aligns with circular economy principles by maximizing value recovery from a single raw material stream.

On the application side, extrusion technology is gaining prominence as a means of enhancing the techno–functional properties of high-fiber BSG, enabling its incorporation into snacks and meat analogues without compromising texture [176,177,178]. In bakery systems, strategies involving particle size adjustment, hydrocolloid inclusion, and pre-fermentation have maintained or improved bread quality at inclusion levels up to 20% BSG, mitigating the volume loss and crumb hardening often reported at higher concentrations [129].

Beyond food, BSG-derived phenolic extracts have been successfully incorporated into active packaging films for controlled antioxidant release [179] and fermentation-derived BSG fractions are being explored for cosmeceutical applications due to their skin-protective bioactivity [180]

Regarding sensory challenges, particularly the bitterness and earthy flavors associated with high BSG inclusion, lactic acid bacteria (LAB) fermentation has demonstrated the potential to modify phenolic and peptide profiles, attenuating undesirable notes while enhancing antioxidant potential [181,182] Additionally, metabolic engineering of flavor pathways in LAB has been proposed to generate desirable aroma compounds, such as acetoin, that can mask harsh flavors in BSG-based products [182,183].

Collectively, these advances highlight a transition toward sustainable, multifunctional valorization routes for BSG, where extraction technology is strategically coupled with product formulation and sensory optimization. Future research should focus on scaling up SWE and DES-based systems, validating multi-step biorefineries at an industrial scale, and conducting long-term sensory and consumer acceptance studies for BSG-enriched products across different food categories.

## 5. Conclusions and Future Perspectives

BSG is the main solid by-product of the brewing industry, accounting for approximately 85% of its waste and generating approximately 20 kg for every 100 L of beer produced. This high availability positions it as a strategic resource for recovering high-value compounds, making a tangible contribution to the circular economy and reducing food waste and CO_2_ emissions. Its potential lies in its remarkable nutritional and bioactive composition, which includes up to 70% dietary fiber, 12–31% protein, polysaccharides such as arabinoxylans and β-glucans, phenolic compounds (ferulic and *p*-coumaric acids), and essential minerals such as phosphorus, calcium, and magnesium.

In recent decades, various technologies have been explored to maximize its use, ranging from drying methods for stabilization, such as freeze drying, which preserves thermosensitive compounds, or infrared drying, which optimizes energy use, to enzymatic hydrolysis and supercritical fluid extraction, which can release and concentrate bioactive peptides with antioxidant and immunomodulatory properties.

The incorporation of BSG has proven to be versatile across multiple food matrices, including bakery products, pasta, cookies, muffins, cereal bars, meat products (burgers, sausages, nuggets), and meat analogs. Its addition not only enriches the nutritional profile by increasing the fiber, protein, and mineral content, but also improves technological properties such as texture, stability, and water-holding capacity, while maintaining or even enhancing sensory acceptance when used in controlled proportions (typically below 20%). Furthermore, BSG particle size is a key factor: fine fractions promote higher protein content, whereas coarser particles optimize the dietary fiber contribution.

Nevertheless, large-scale implementation still faces significant challenges. These include the high perishability of fresh BSG, compositional variability depending on raw materials and brewing processes, regulatory requirements, and consumer acceptance, particularly at higher inclusion levels, which may alter flavor or texture. In addition, there is a pressing need for in vivo studies and clinical trials to confirm the health benefits, bioaccessibility, and safety of its compounds under real physiological conditions, as well as to evaluate their behavior in complex food matrices and assess the economic feasibility of industrial-scale processing.

Looking ahead, the valorization of BSG as a functional ingredient is strongly aligned with the United Nations Sustainable Development Goals (SDGs) related to responsible production, industrial innovation, and food security. Its use in meat and hybrid products offers a tangible opportunity to move toward more resilient, inclusive, and circular food systems. However, consolidating this “silent revolution” will require a multidisciplinary approach integrating science, technology, sustainability, and effective consumer communication.

Strengthening collaborations among the brewing industry, food sector, research centers, and regulatory bodies will be essential to overcome technical and legal barriers and establish safe, economically viable, and environmentally responsible production models. In this scenario, technological advances in drying, controlled fermentation, enzymatic hydrolysis, and assisted extraction will continue to play a central role, not only in preserving and enhancing BSG’s functional value but also in expanding its applications to new market niches.

## Figures and Tables

**Table 1:** Advantages and limitations of the technologies used in the extraction of BSG.

Technology	Target Compounds	Advantages	Limitations
Conventional Solid–Liquid Extraction	Phenolic compounds, lipids	Simple, well-established, relatively low cost; suitable for various solvents (e.g., ethanol, methanol).	Low selectivity; long extraction times; possible degradation of heat-sensitive compounds; high solvent use and disposal concerns.
Microwave-Assisted Extraction	Phenolics, proteins, polysaccharides	High extraction efficiency in a short time; reduced solvent use; effective cell wall disruption.	Non-uniform heating can reduce reproducibility; high equipment cost; limited scalability.
Ultrasound-Assisted Extraction	Phenolics, polysaccharides, proteins	Enhance mass transfer; low temperature process preserves bioactivity; relatively low cost.	Possible degradation of sensitive compounds due to cavitation; scalability challenges for uniform energy distribution.
Enzymatic Hydrolysis	Proteins (bioactive peptides), polysaccharides (β-glucans, arabinoxylans)	Highly selective; mild processing preserves functional properties; generates high-purity extracts.	High cost of enzymes; requires precise control of pH and temperature; potential long processing times.
Alkaline Hydrolysis	Bound phenolics, proteins	Efficient for breaking ester bonds and releasing bound phenolics; relatively inexpensive.	May degrade sensitive bioactives; environmental concerns from alkaline waste; reduced suitability for food applications without purification.
Supercritical Fluid Extraction	Lipids, non-polar compounds	Solvent-free extracts; preserves thermo-labile compounds; environmentally friendly; tunable selectivity via pressure/temperature.	High capital and operating costs; not ideal for highly polar compounds without co-solvents.
Pressurized Hot Water Extraction (PHWE/Subcritical Water Extraction)	Phenolics, polysaccharides	High efficiency; avoids organic solvents; eco-friendly; effective release from lignocellulosic matrix.	Requires high-pressure, high-temperature equipment; risk of thermal degradation of heat-sensitive compounds.
Deep Eutectic Solvent Extraction	Phenolics, polysaccharides	Green, biodegradable solvents; high solubility for phenolics; potential to replace hazardous solvents.	Limited industrial validation: viscosity of solvents may hinder large-scale processing.
Infrared Drying + Milling Pre-treatment	General compounds (preparation step)	Reduces drying time and energy; preserves aroma and color; improves downstream extraction efficiency.	Requires investment in specialized equipment; it may not be suitable for all bioactives.

**Table 2:** Incorporation of BSG in meat and analog products.

Type of Product Analyzed	BSG Onboarding	Functionality Technology	Main Finding	Author
Sausage Hybrids	Partial meat substitution (35%) with BSG, broccoli and insects	Modeling with simplex design and optimization by desirability function	Protein enhancement, traditional sausage-like texture, sensory acceptance	[164]
Cuiabana sausage	Partial replacement of meat with BSG meal (up to 6%)	Physical–chemical and microbiological analysis	Increased fiber and protein, no lipid oxidation	[163]
Burgers	Fat replacement with malt bagasse (up to 3%)	Antioxidant evaluation and texture	Higher fiber, lower calories, improved cooking parameters	[162]
Frankfurters	BSG Fat replacement	Textural and compositional analysis	Improved texture and fat reduction without affecting quality	[165]
Fish burgers	Microencapsulation of BSG bioactive compounds	Bioactive enrichment	Increase in bioactive compounds without altering sensory properties	[166]
Low-fat chicken sausages	Replacing Fat with BSG Dietary Fiber	Textural and nutritional profile analysis	Better nutritional quality and texture in reduced-fat products	[167]
Chicken burgers	Addition of BSG Dietary Fiber	Convective oven cooking	Better moisture retention and firm texture	[159]
Vegetable meat analogues	Incorporation of BSG as an ingredient (up to 20%)	Extrusion in vegetable mixtures	Improved fibrous texture and nutritional profile (protein and fiber)	[168]
Extruded snacks	Mixing BSG with poultry meal	High temperature extrusion	Snacks rich in fiber and protein, acceptable for consumption	[155]
Bean meat analogues	Addition of enzymatically treated BSG	Texture improvement using enzymes	More meat-like structure, better cohesion and bite	[169]
Enriched burgers	BSG as a source of fiber (3.6%)	Sustainability Perception Survey	Increased acceptance when communicating environmental benefits	[170]
Traditional cooked sausage	Addition of BSG (2–4%)	Stability analysis during storage	Maintenance of physicochemical quality and shelf life of 21 days	[156]
Chicken Nuggets	Using BSG as a High-Fiber Breader	Oil Absorption Evaluation	Reduction in absorbed oil, higher nutritional quality	[157]
